# Feasibility of community-based screening for cardiovascular disease risk in an ethnic community: the South Asian Cardiovascular Health Assessment and Management Program (SA-CHAMP)

**DOI:** 10.1186/1471-2458-13-160

**Published:** 2013-02-21

**Authors:** Charlotte A Jones, Alykhan Nanji, Shefina Mawani, Shahnaz Davachi, Leanne Ross, Ardene Vollman, Sandeep Aggarwal, Kathryn King-Shier, Norman Campbell

**Affiliations:** 1 Department of Medicine, University of Calgary, Libin Cardiovascular Institute, TRW Building GE89, 3280 Hospital Drive NW, Calgary, AB, Canada; 2 Libin Cardiovascular Institute, Calgary, AB, Canada; 3 University of Calgary, Calgary, AB, Canada; 4 Alberta Health Services, Calgary, AB, Canada; 5 Faculty of Nursing, University of Calgary, Calgary, AB, Canada

**Keywords:** Community-based participatory research, Ethnic groups, Health status disparities, Hypertension, Cholesterol

## Abstract

**Background:**

South Asian Canadians experience disproportionately high rates of cardiovascular disease (CVD). The goal of this qualitative study was to determine the feasibility of implementing a sustainable, culturally adapted, community-based CVD risk factor screening program for this population.

**Methods:**

South Asians (≥ 45 years) in Calgary, Alberta underwent opportunistic cardiovascular risk factor screening by lay trained volunteers at local religious facilities. Those with elevated blood pressure (BP) or ≥ 1 risk factor underwent point of care cholesterol testing, 10-year CVD risk calculation, counseling, and referral to family physicians and local culturally tailored chronic disease management (CDM) programs. Participants were invited for re-screening and were surveyed about health system follow-up, satisfaction with the program and suggestions for improvement. Changes in risk factors from baseline were estimated using McNemar’s test (proportions) and paired t-tests (continuous measures).

**Results:**

Baseline assessment was completed for 238 participants (median age 64 years, 51% female). Mean TC, HDL and TC/HDL were 5.41 mmol/L, 1.12 mmol/L and 4.7, respectively. Mean systolic and diastolic blood pressures (mmHg) were 129 and 75 respectively. Blood pressure and TC/HDL ratios exceeded recommended targets in 36% and 58%, respectively, and 76% were at high risk for CVD. Ninety-nine participants (47% female) attended re-screening. 82% had accessed health care providers, 22% reported medication changes and 3.5% had attended the CDM programs. While BP remained unchanged, TC and TC/HDL decreased and HDL increased significantly (mean differences: -0.52 mmol/L, -1.04 and +0.07 mmol/L, respectively). Participants were very satisfied (80%) or satisfied (20%) with the project. Participants suggested screening sessions and CDM programs be more accessible by: delivering evening or weekends programs at more sites, providing transportation, offering multilingual programs/translation assistance, reducing screening wait times and increasing numbers of project staff.

**Conclusions:**

SA-CHAMP demonstrated the feasibility and value of implementing a lay volunteer–led, culturally adapted, sustainable community-based CVD risk factor screening program in South Asian places of worship in Calgary, Alberta, Canada. Subsequent screening and CDM programs were refined based on the learnings from this study. Further research is needed to determine physician and patient factors associated with uptake of and adherence to risk reduction strategies.

## Background

While cardiovascular disease (CVD) rates in Canada have decreased substantially in recent decades, significant disparities persist among certain ethnic groups. Along with Aboriginal populations, Canadian South Asians (SAs) experience CVD morbidity and mortality rates 2 to 5 times higher than those of European or Chinese descent [1-5]. Furthermore, both the INTERHEART [6] and a recent study done in Canada [3] suggest that SAs tend to have their first myocardial infarctions about 5 years earlier, on average, relative to the general populations in Canada and elsewhere. These findings mirror those of epidemiological studies from South Africa, Trinidad, Singapore, Fiji, Mauritius, the United States and the UK (reviewed in: [7,8]), suggesting that SAs in these countries also carry a disproportionate CVD burden compared with the general population.

Modifiable contributors to this disparity in SA’s include the excessively high (3–5 times higher) prevalence of diabetes (including poorly controlled/undetected diabetes), and metabolic syndrome with dyslipidemias including low HDL-cholesterol and high triglycerides relative to the general populations in these countries [1,7,9-13]. Additional significant contributors to these observed disparities include language difficulties, low health literacy, decreased medication adherence, disparate health beliefs, and lack of knowledge, understanding and appreciation of the serious nature of CVD [14-17].

Compared with general public education and social marketing strategies, better health outcomes result from interventions that assess individual risk, encourage personal risk reduction, and put resources in place that create a supportive environment for health and strengthen community action [18-21]. Extensive review of the world literature suggests that community-based participatory research (CBPR) methods that tailor interventions to target barriers specific to a minority group have the greatest potential to reduce disparities in care and outcomes [22-30]. Studies undertaken with participants in their own communities, particularly faith-based interventions involving community health workers or lay health volunteers and the use of educational materials specially adapted for the language, culture and literacy needs of the minority group, showed the greatest promise.

Many exemplar CVD screening and intervention programs originating in Canada, [31,32] the United States [25,28,33-35], the United Kingdom [36-38], India [39] and Pakistan [40] have employed a variety of personnel (peer educators, community health workers, trained barbers, medical/paramedical volunteers) in religious settings [19,20,41-45] to improve cardiovascular health among various minority groups, including African Americans, Hispanics, older adults and low income women. However, short of a similar diabetes program [46], to the best of our knowledge, global CVD risk screening and education programs for SAs that are led by trained lay community volunteers in religious settings have not been implemented in Canada.

SAs constitute the second largest visible minority population in Canada after those with Chinese origins. Calgary, Alberta has the third largest visible minority population in Canada. In 2006, there were over 1.3 million people of SA ethnicity in Canada; over 60,000 of who lived in Calgary [47]. Considering the multiple medical and socio-cultural barriers limiting participation in mainstream programs, accessible and culturally appropriate CVD prevention, screening and risk reduction programs for SAs in Canada are needed.

The objective of this primarily qualitative study was to determine the feasibility of implementing a sustainable, culturally adapted, lay volunteer led community-based CVD risk factor screening program located within places of worship in the SA (originating from Bangladesh, India, Pakistan, and Sri Lanka) community in Calgary, Alberta.

## Methods

### The setting

University of Calgary researchers were approached by SA community leaders in Calgary, Alberta to implement a program similar to two local projects that addressed CVD risk reduction [32,46]. After receiving ethics approval (from the Calgary’s Conjoint Health Research Ethics Board) and drawing up a memorandum of understanding, six religious facilities were chosen by these same community leaders as screening locations. Participation was invited by community leaders through weekly announcements at the facilities and notices in local community newsletters, radio and television.

### Family physicians

Family physicians adjacent to the screening venues were invited by letter to a group information and workshop session. Attendees were provided an overview of the study and an update on the Canadian guidelines for hypertension management [48]. Study staff (SM, CAJ, AN) contacted and discussed the program with physicians who did not attend.

### Volunteer training

Forty-nine SA lay community volunteers and allied health care professionals (2 nurses, 2 dieticians, 2 pharmacists and 5 medical students) as identified by community leaders, were provided with a modified version of a validated culturally adapted volunteer training tool [46] to read and attended two 3-hour group training sessions [32]. Volunteers were trained to assess CVD risk and provide advice, education, family physician referral, and referral to local culturally tailored chronic disease management (CDM) programs. Volunteers were provided with information on CVD and major risk factors (unhealthy eating, physical inactivity, smoking, excess alcohol consumption, hypertension, dyslipidemia and diabetes). Training in blood pressure (BP) measurement (BpTRU device (model BPM 100^®^:VSM MedTech LTD) and random capillary assessment of total and HDL cholesterol (Cholestech^®^ desk top reflometer, Hayward, CA., USA.) was delivered by a certified trainer from the Calgary Fire Department [49].

Volunteers were trained to assess 10-year total CVD risk using the British Hypertension Society CVD Risk Prediction Chart [50] (http://www.bhsoc.org/Cardiovascular_Risk_Prediction_Chart.stm). As this risk assessment tool underestimates risk among SAs, scores were multiplied by a factor of 1.5 [51,52]. While this risk assessment tool has not been validated in SAs (nor was any other at the time of this study), it was chosen because we were interested in assessing total CVD risk rather than just coronary heart disease risk (as in the Framingham risk tool available at the time of the study [53]) and the simple colored graphics were deemed user friendly for our lay volunteer group.

University of Calgary, Faculty of Medicine experts provided clinical expertise and training; a project coordinator from the community worked with researchers to manage the volunteers and screening sessions.

### Baseline screening

SAs 45 years and older (non-pregnant) presenting for screening were greeted by a project volunteer who confirmed their language of choice and directed then to the appropriate volunteer for eligibility assessment. All program activities were delivered in the participant’s language of choice (English, Gujarati, Punjabi, Hindi or the Dari dialect).

After obtaining signed ethics consent, brief questionnaires assessing personal history of CVD, family history of premature CVD, smoking status, and presence of known hypertension, hypercholesterolemia and/or diabetes were administered. Individuals self-reporting a physician diagnosis of or use of medication(s) for high blood pressure, high cholesterol and/or high blood sugar were considered to have hypertension, hypercholesterolemia and/or diabetes, respectively.

Resting BP was measured as per the Canadian guidelines [48] using the automated BPTru^®^ monitor. After five minutes rest, six readings were obtained at one minute intervals. The first reading was discarded and the average of the last five was recorded. Those with elevated systolic BP (≥ 140 mm Hg or ≥ 130 mm Hg among individuals with diabetes) and/or elevated diastolic BP (≥ 90 mm Hg or ≥ 80 mm Hg among those with diabetes) or 1 or more CVD risk factor (personal history of CVD, family history of premature CVD, self reported diabetes, hypertension or dyslipidemia, current smoking) were eligible for further study. Eligible participants underwent non-fasting testing for total cholesterol/high density lipoprotein (TC/HDL) ratio and calculation of 10-year total CVD risk. Risk was classified as low-moderate (≤ 20%) or high (> 20%). Those with self-reported CVD or diabetes were automatically classified as high risk. The TC/HDL ratio was considered within target if < 4 (high risk participants) or < 6 (low-moderate risk participants) [54].

Screening results and culturally adapted educational materials [55] detailing CVD risk factors, diet, physical activity, alcohol and smoking were reviewed with and then given to participants. Participants were asked to follow up with their family physicians within one month of screening. Copies of their assessments were faxed to their family physicians, with the suggested option of referring high risk participants to one of three local, culturally sensitive CVD assessment clinics. Low-moderate risk participants were asked to self-refer to the health region’s CDM program, consisting of culturally tailored lifestyle counseling and exercise programs. Community leaders insisted that all participants receive the same advice and follow-up recommendations at the same time, precluding any form of randomization (e.g., to immediate vs. delayed intervention).

### Follow-up screening

After 6–13 months (median = 9 months), participants who completed baseline assessments were contacted by telephone and invited for follow-up. Study funding limited re-screening to the first 100 consecutive participants. They were re-screened exactly as above, and then completed a questionnaire assessing reasons for joining the study; follow-up with family physicians, high risk clinics or CDM programs; satisfaction with the study and suggestions for improvement.

### Analysis

Changes in BP and cholesterol levels (follow-up minus baseline) were calculated and average differences (with 95% confidence intervals) were estimated using McNemar’s test (proportions) and paired t-tests (continuous variables). Change was also assessed by subgroup of interest: gender and self-reported diabetes. All analyses were done using Stata12 (College Stn, TX).

## Results

### Baseline screening

Between May 30 and November 15, 2007, 374 participants attended 12 three hour screening sessions held in 6 different places of worship. Fifty were excluded: < 45 years of age (n = 5), age unknown (n = 5), declined BP measurement (n = 19), declined further participation after completing health history (n = 13), and currently under the care of a cardiac specialist (n = 8). Two hundred thirty eight were eligible for complete screening.

Men and women were equally represented and had similar age distributions (Table 1). The majority selected English as their language of choice. Thirty-six percent had elevated BP, 58% had elevated TC/HDL ratios, 23% reported diabetes and 76% were at high risk for CVD. Compared with women, men were significantly more likely to have elevated TC/HDL ratios (78% vs. 40%) and to be at high risk for CVD (94% vs. 59%). Those with diabetes were more likely than non-diabetics to have hypertension (measured plus reported) and elevated TC/HDL ratios (data not shown).

**Table 1:** Demographic and baseline screening information for SA-CHAMP participants

**Characteristic**	**All**	**Female**	**Male**
	**N = 238**	**N = 122**	**N = 116**
	**n (%)**	**n (%)**	**n (%)**
Age (years)			
45–59	81 (34)	36 (30)	45 (39)
60–79	142 (60)	79 (65)	63 (54)
80 or older	15 (6)	7 (6)	8 (7)
Program language of choice			
English	170 (71)	82 (67)	88 (76)
Gujarati	41 (17)	31 (25)	10 (9)
Punjabi	20 (8)	2 (2)	18 (16)
Dari	4 (2)	4 (3)	-
Hindi	3 (1)	3 (3)	-
Reported history of CVD^*^	24 (10)	9 (7)	15 (13)
Reported history of stroke/TIA^†^	5 (2)	1 (1)	4 (4)
Parent or sibling with myocardial infarction or stroke before age 60	67 (28)	37 (30)	30 (26)
Current smoker or quit within 3 months	11 (5)	-	11 (10)
Reported history of hypercholesterolemia	116 (49)	54 (44)	62 (54)
Reported history of diabetes	55 (23)	28 (23)	27 (23)
Reported history of hypertension	124 (52)	70 (57)	54 (47)
Elevated systolic and/or diastolic blood pressure^‡^	86 (36)	47 (39)	39 (34)
Controlled TC/HDL ^§^	99 (42)	73 (60)	26 (11)
Estimated 10-year CVD risk			
low-moderate (<=20%)	57 (24)	50 (41)	7 (6)
high (>20%)	181 (76)	72 (59)	109 (94)
	**mean (SD)**	**mean (SD)**	**mean (SD)**
	**range**	**range**	**range**
Systolic blood pressure (mmHg)	129 (18.9)	131 (18.8)	127 (18.9)
	94-185	97-184	94-185
Diastolic blood pressure (mmHg)	75 (10.4)	74 (9.6)	77 (11.1)
	50-116	53-96	50-116
Total cholesterol (mmol/L)	5.41 (1.1)	5.57 (1.2)	5.25 (1.1)
	2.59-9.91	2.85-9.91	2.59-9.37
HDL (mmol/L)	1.12 (0.35)	1.23 (0.33)	0.99 (0.33)
	0.39-2.06	0.47-2.06	0.39-2.03
	**median (IQR**^**¶**^**) range**	**median (IQR**^**¶**^**) range**	**median (IQR**^**¶**^**) range**
TC/HDL	4.7 (3.8-6.5)	4.4 (3.5-5.4)	5.3 (4.2-7.1)
	2.4-16	6 2.4-14.0	2.5-16.7

* CVD: cardiovascular disease.

† TIA: transient ischemic attack.

^‡^ Elevated systolic blood pressure: > = 140 mm Hg (non-diabetic) or > = 130 mm Hg (diabetic); elevated diastolic blood pressure: > = 90 mm Hg (non-diabetic) or > = 80 mm Hg (diabetic).

^§^ Controlled TC/HDL: < 4 for high risk participants; < 6 for low-moderate risk participants.

^¶^ IQR: interquartile range.

### Follow-up screening

Of the 100 individuals who completed follow-up, 1 did not meet the original study eligibility criteria and was excluded. While comparable on all other variables (data not shown), individuals presenting for follow-up had a higher median TC/HDL ratio when compared with all who presented for baseline screening (5.2 vs. 4.7).

Mean systolic and diastolic BP did not change from baseline to follow-up (mean differences: -0.3 mm Hg and −0.5 mm Hg, respectively) and remained elevated in 35% at follow-up (Table 2). The average total cholesterol decreased significantly overall and among all subgroups examined while the average HDL increased overall and among all subgroups examined with the exception of those with diabetes in whom the average HDL did not change. The average TC/HDL ratio decreased significantly overall and among all subgroups examined. While a significant increase in the proportion of individuals with TC/HDL ratios within target was noted overall, the improvement appeared to be limited to men (18%) and those without diabetes (14%) (Table 2).

**Table 2:** Change in blood pressure and TC/HDL among SA-CHAMP participants who completed follow-up

**Characteristic**	**n**	**Baseline proportion**	**Follow-up proportion**	**Mean difference (95% C.I.)**	**p-value**
Elevated systolic and/or diastolic blood pressure ^*^					
All	99	0.39	0.35	−0.04 (−0.14 to 0.06)	0.524
Men	52	0.38	0.33	−0.06 (−0.21 to 0.10)	0.581
Women	47	0.40	0.38	−0.02 (−0.17 to 0.12)	1.000
Diabetics	27	0.52	0.56	0.04 (−0.22 to 0.30)	0.739
Non-diabetics	72	0.35	0.28	−0.07 (−0.18 to 0.04)	0.267
Controlled TC/HDL^§^					
All	98	0.34	0.46	0.12 (0.01 to 0.23)	0.029
Men	51	0.20	0.37	0.18 (0.03 to 0.33)	0.023
Women	47	0.49	0.55	0.06 (−0.10 to 0.23)	0.581
Diabetics	27	0.30	0.37	0.07 (−0.14 to 0.29)	0.688
Non-Diabetics	71	0.35	0.49	0.14 (0.01 to 0.27)	0.041
	**n**	**Mean at baseline**	**Mean at follow-up**	**Mean paired difference (95% C.I.)**	**p-value**
Total Cholesterol					
All	99	5.56	5.04	−0.52 (−0.71 to −0.34)	< 0.001
Men	52	5.34	4.81	−0.53 (−0.81 to −0.24)	< 0.001
Women	47	5.81	5.30	−0.52 (−0.76 to −0.28)	<0.001
Diabetics	27	5.45	5.13	−0.32 (−0.63 to −0.01)	0.043
Non-Diabetics	72	5.61	5.01	−0.60 (−0.83 to −0.37)	<0.001
HDL Cholesterol					
All	98	1.08	1.15	0.07 (0.03 to 0.11)	0.002
Men	51	0.96	1.03	0.06 (0.00 to 0.12)	0.038
Women	47	1.21	1.29	0.08 (0.01 to 0.14)	0.029
Diabetics	27	1.08	1.11	0.04 (0.06 to 0.13)	0.456
Non-Diabetics	71	1.09	1.17	0.08 (0.03 to 0.13)	0.002
TC/HDL					
All	98	5.80	4.76	−1.04 (−1.41 to −0.68)	<0.001
Men	51	6.34	5.03	−1.31 (−1.92 to −0.69)	<0.001
Women	47	5.21	4.46	−0.76 (−1.13 to −0.38)	<0.001
Diabetics	27	5.85	4.98	−0.87 (−1.70 to −0.04)	0.041
Non-Diabetics	71	5.78	4.67	−1.11 (−1.51 to −0.71)	<0.001

* Elevated systolic blood pressure: > = 140 mm Hg (non-diabetics) or > = 130 mm Hg (diabetics); elevated diastolic blood pressure: > = 90 mm Hg (non-diabetics) or > = 80 mm Hg (diabetics).

^§^ Controlled TC/HDL: < 4 for high risk participants; < 6 for low-moderate risk participants.

All 99 participants completed the evaluation questionnaire. Reasons for taking part in the study included interest in knowing their BP and cholesterol (35%), desire for follow-up of a pre-existing health concern (31%) or to obtain more information about cardiovascular health (22%), curiosity (18%) and being asked to attend by someone else (18%). Fifty-eight percent reported being somewhat or a lot worried by the information that they received. Most (82%) had visited their family physicians to discuss their results, with no difference by age, sex, or CVD risk category. Among these, 66% discussed their CVD risk profiles and 22% had medication changes initiated. Specialty clinic referrals were made for 31% of high risk participants; 84% attended, 10% did not, and 6% were awaiting appointments. A search of the CDM program registry revealed that only 2 of the 57 low-moderate risk participants had self-referred to the available programs. Time constraints (44%) and lack of perceived need (9%) were the two most commonly cited reasons for non-attendance.

While all participants reported being very satisfied (80%) or satisfied (20%) with the project, it was suggested that screening sessions and CDM programs be made more accessible by: delivering programs during evening hours or weekends and at more sites; providing transportation; offering multilingual programs or translation assistance; reducing screening wait times and increasing the numbers of project staff.

Since completion of this program, and based on our participants’ suggestions, local CDM programs are now being offered at more sites and with extended hours of operation. Furthermore, CVD risk screenings have continued in many of the participating SA communities and have been refined based on the suggestions made by our SA CHAMP participants. A community volunteer maintains the program and has trained several other volunteers. With the Public Health Agency of Canada’s support, similar programs piloting screening coupled with “health buddy” follow-up are ongoing in Calgary and five other cities across Canada.

## Discussion

The SA-CHAMP is unique in that it demonstrated the feasibility of using trained lay community volunteers to run a sustainable, culturally adapted opportunistic CVD risk factor screening project in places of worship within the SA community in Calgary, Alberta, Canada. Furthermore, despite this being a pilot trial, modest but significant improvement in cholesterol measures were noted. Much of the success and acceptability of this program is a reflection of the high level of community engagement and volunteer participation throughout all stages of the program including the design, implementation and evaluation; key factors in the well accepted CBPR approach to reducing health disparities in minority populations [22]. The degree of community “buy-in” or ownership of the program and the strong commitment of the lay community members that has led to the dissemination of the program beyond its original setting to SA communities across Canada also makes this program unique in Canada.

Places of worship play a strong cultural and social role in many communities across the world, and are a place where community members come together. They have been shown to provide a particularly relevant and culturally comfortable setting for health-related interventions [19,43,44,56,57]. The bulk of this evidence is derived from studies done in the United States within African American and Hispanic communities. However, there are two recent studies other than ours suggesting the feasibility of using places of worship in SA communities to identify individuals with CVD risk factors. Davachi et al. [46] demonstrated that when diabetes screening sessions for those without known diabetes were held in the context of religious gatherings in temples and mosques in Calgary, Alberta, it was highly acceptable to community members and is reflected in the high participation rate (922 screened in 14 four hour screening sessions). Similarly, Rao et al. [45] screened for CVD risk in two Hindu temples in London, England. In this study medical/paramedical community members were able to screen 434 self-selected participants in 9 six-hour sessions. This group is currently carrying out a qualitative assessment of the acceptability of the program among staff and participating community members.

Use of trained lay health workers as part of the health-care team in community-based program settings is recognized as a way to improve access and the health of underserved populations [22]. Community members trained as peer health advisors, particularly those who are members of the target community, may play a significant role in addressing cardiovascular risk factors because of their connectedness to individuals and communities, as well as their understanding of cultural and contextual issues [28,58,59]. A noteworthy example is the recent study looking at the effectieveness of barbers who were trained and paid to become health educators, monitor BP, and promote physician referral for patrons of local African-American-owned barbershops in Dallas County, Texas, USA. While improvement in the primary outcome of hypertension among African-American male patrons was modest (8.8%, 95% confidence interval: 0.8%-16.9%), 98% of participants and all 29 participating barbers reported that they would like the program continued indefinitely.

Until recently, there has been a paucity of literature on the effectiveness of trained lay community members as volunteers, specifically in the context of community-based CVD risk factor screening and improved CVD outcomes. In a cluster randomized controlled trial of 39 communities in Ontario, Canada [31], volunteer lay seniors were trained to perform BP and cardiovascular risk assessment on other seniors invited by their physicians to attend pharmacy-based screening clinics. Within one year of the screening program, a 9% relative reduction in CVD-related hospital admissions was noted, translating to 3.02 fewer hypertension-related hospitalizations per 1000 people aged 65 and older in the intervention communities. Our study and that of Davachi et al. [46] differ from the Kaczorowski study in that they were both adapted for the SA community and as such, the lay trained volunteers were SA and the screenings took place in religious facilities rather than community pharmacies. The use of trained lay community volunteers in this setting shows promise and our study helps to support the feasibility of implementing a similar larger RCT in SA communities to evaluate whether such an intervention can reduce CVD in this group.

The value (in terms of identifying at-risk individuals that would benefit from intervention) of opportunistic CVD risk screening in SA places of worship has not been well described. Using a methodology similar to ours, Davachi et al. [46] opportunistically screened adult non-diabetic SA for diabetes in places of worship, and observed very high rates of obesity (67%), family history of diabetes (43%) and results suggestive of potential glucose intolerance (36%). Despite excluding of those with self reported CVD, hypertension, diabetes or use of lipid therapy, [45] Rao et al. found that 52% of participants had hypertension, 75% had central obesity, 10% had TC/HDL ratios > 6, and 15% were assessed as high risk using the QRISK2 model for 10-year CVD risk. The higher prevalence of both poorly controlled risk factors and 10-year CVD risk among participants in our study are consistent with their higher average age and their relatively high prevalence of self-reported CVD (10%), diabetes (23%), hypertension (52%) and dyslipidemia (49%). While these three studies are not directly comparable given the variable inclusion criteria, screening techniques, ages and ethnic groups represented, a high prevalence of modifiable, poorly controlled CVD risk in the SAs that self-presented for screening was a consistent finding. The majority of participants were at moderate to high risk for CVD and therefore represented an ideal target group for improving awareness and risk factor management. Further, participants in our study were satisfied with the program and barriers to healthcare provider access did not appear to be a problem. Overall, these three studies provide support for the feasibility, acceptability and value of screening SA adults for CVD risk factors in places of worship.

Our study has limitations. Participants self-selected for study and it is not known if participants in this study are representative of the SA community at large. Self-selected participants may have been more motivated, particularly those who returned for follow-up, which could partially explain the observed reduction in TC/HDL ratios and high rates of follow-up with family physicians. Our study design (single group, before-and-after) was also vulnerable to regression to the mean. While significant TC/HDL ratio reductions were still observed when the analysis was limited to those without outlying values at baseline, random variation, rather than follow-up care, may explain some of the observed improvements in lipid control. Furthermore, while there are small variations in lipid measures between the Cholestech^®^ desk top reflometer and full laboratory-based lipid profiles, especially in the case of elevated triglycerides, Cholestech^®^ measures are considered acceptable under these circumstances [60]. However, we cannot rule out such variability as contributing a measure of uncertainty to our cholesterol measures. Additionally, relying on a single measure of blood pressure at baseline may have led to an overestimation of the prevalence of hypertension among those screened.

With respect to data collection, we had no way of validating self-reported information. The validity of self-report of CVD risk factors and follow-up behavior in the context of reporting such sensitive information to a community volunteer possibly known to the participant is an interesting area for future study. Further, the study protocol depended to a great extent on trained lay volunteers and may not be generalizable to all SA communities where volunteerism is not as prevalent.

Given that this study was primarily qualitative, and that no a priori power/sample size calculation was performed, the significance of the pre-post changes (or lack thereof) in clinical parameters should not be over-interpreted. These analyses were conducted on an exploratory basis only. The variability in the studies measures will be used to determine a sample size for a future, larger study.

Finally, while modified versions of the program have been initiated in Calgary and 5 other cities in Canada, it is not yet known if the programs will prove feasible on a large and sustainable scale and demonstrate effectiveness within the target population.

## Conclusions

SA-CHAMP demonstrated the feasibility of implementing a culturally adapted, sustainable community-based CVD risk factor screening program in the SA community in Calgary, Alberta, Canada. SA-CHAMP identified a high risk cohort amenable to intervention. Participants’ input has helped to refine the next iteration of screening programs and has helped change policy for the delivery of local CDM programs. Further research is needed to determine the content and delivery of sustainable intervention programs that will successfully reduce CVD risk factors and disease risk in this population.

## Abbreviations

SA-CHAMP: South Asian Cardiovascular Health Assessment and Management Program; TC/HDL: Total Cholesterol/High Density Lipoprotein; CVD: Cardiovascular Disease; BP: Blood Pressure.

## Competing interests

The authors’ declare that they have no competing interests.

## Authors' contributions

CAJ conceived of the study, participated in its design and coordination and drafted the manuscript. LMR performed the statistical analysis and participated in drafting the manuscript. All authors read and approved the final manuscript.

## Pre-publication history

The pre-publication history for this paper can be accessed here:

http://www.biomedcentral.com/1471-2458/13/160/prepub
